# Characterization of Active Electrode Yield for Intracortical Arrays: Awake versus Anesthesia

**DOI:** 10.3390/mi13030480

**Published:** 2022-03-20

**Authors:** Brandon Sturgill, Rahul Radhakrishna, Teresa Thuc Doan Thai, Sourav S. Patnaik, Jeffrey R. Capadona, Joseph J. Pancrazio

**Affiliations:** 1 Department of Bioengineering, The University of Texas at Dallas, 800 W. Campbell Road, Richardson, TX 75080, USA; brandon.sturgill@utdallas.edu (B.S.); radhakrishnar@utdallas.edu (R.R.); teresa.thai@utdallas.edu (T.T.D.T.); sourav.patnaik@utdallas.edu (S.S.P.); 2 Department of Biomedical Engineering, Case Western Reserve University, Advanced Platform Technology Center, Louis Stokes Cleveland Veterans Affairs Medical Center, Cleveland, OH 44106, USA; jrc35@case.edu

**Keywords:** anesthesia, microelectrode arrays, single-unit activity, bursts, cortex

## Abstract

Intracortical microelectrode arrays are used for recording neural signals at single-unit resolution and are promising tools for studying brain function and developing neuroprosthetics. Research is being done to increase the chronic performance and reliability of these probes, which tend to decrease or fail within several months of implantation. Although recording paradigms vary, studies focused on assessing the reliability and performance of these devices often perform recordings under anesthesia. However, anesthetics—such as isoflurane—are known to alter neural activity and electrophysiologic function. Therefore, we compared the neural recording performance under anesthesia (2% isoflurane) followed by awake conditions for probes implanted in the motor cortex of both male and female Sprague-Dawley rats. While the single-unit spike rate was significantly higher by almost 600% under awake compared to anesthetized conditions, we found no difference in the active electrode yield between the two conditions two weeks after surgery. Additionally, the signal-to-noise ratio was greater under anesthesia due to the noise levels being nearly 50% greater in awake recordings, even though there was a 14% increase in the peak-to-peak voltage of distinguished single units when awake. We observe that these findings are similar for chronic time points as well. Our observations indicate that either anesthetized or awake recordings are acceptable for studies assessing the chronic reliability and performance of intracortical microelectrode arrays.

## 1. Introduction

Intracortical microelectrode arrays (MEAs) are promising tools for studying brain function and the development of neuroprosthetic strategies. However, improving the chronic performance of these devices remains a major research challenge. Potential failure mechanisms and the corresponding approaches to improve reliability and performance are varied [1,2]. Failure of the microelectrode-array-based neural devices can stem from multiple biotic or abiotic factors. The trauma from insertion as well as the continued presence of these devices elicit a foreign body response, resulting in macrophage and microglia activation, glial encapsulation, neuronal dieback, a breakdown of the blood–brain barrier [3,4], and oxidative stress [5] in the vicinity of the probe. Resulting degradation to the probe and adverse effects on the tissue cause an eventual decrease in long-term recording performance and reliability [4,6,7]. There are many approaches to mitigate the detrimental effects leading to MEA failure. These approaches include decreased probe size [4,8,9], minimizing mechanical mismatch micromotion between the probe and tissue [10,11,12,13], decreasing inflammation and avoiding detection to minimize the foreign body response [14,15,16], and mitigating oxidative stress [17]. 

Metrics to assess chronic MEA reliability include the active electrode yield (AEY), i.e., the percentage of microelectrode sites that exhibit at least one discernable single unit, the signal-to-noise ratio (SNR), signal amplitude, and noise level. An important metric is AEY, which typically decreases significantly over implantation periods of several months [7]. In vivo experiments are the common method of testing the reliability and performance of these devices; however, the recording conditions vary among studies. For example, recordings may be of spontaneous or stimulated activity in anesthetized animals or from animals that are awake and moving about freely or performing a specific task. 

Although recording conditions are varied, anesthetizing implanted animal subjects to facilitate connection to a tethered head-stage during weekly or bi-weekly recording sessions is common [10,11,18]. However, anesthetics do alter electrophysiological function. Anesthetics can decrease the firing rate [19] and suppress burst firing in a dose-dependent manner [20]. Given that anesthetics are known to alter electrophysiological function [21], we asked whether or not there are differences in recording metrics, particularly AEY and SNR, in rats implanted with MEAs in the motor cortex while anesthetized with isoflurane or awake and freely moving. Our observations indicate that both anesthetized and awake recordings yield similar metrics when assessing MEA performance and reliability. While the single-unit firing rate was much greater under the awake recording conditions than the anesthetized conditions, isoflurane anesthesia did not adversely affect the recording metrics important to measuring device reliability and performance when recording from the motor cortex.

## 2. Materials and Methods

### 2.1. Surgical Implantation of Neural Devices

**Figure 1:**
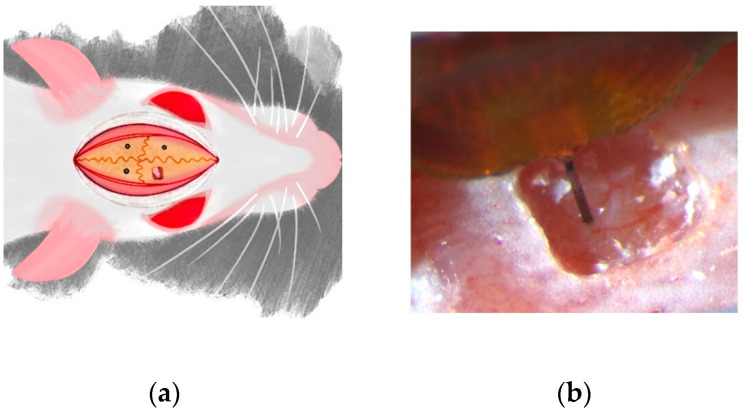
Surgical implantation of neural probes and neurophysiological data acquisition. (**a**) Black dots represent the site of bone screw placement, and the craniotomy was performed in the upper right quadrant defined by ~2 mm anterior to bregma and ~2 mm lateral central suture line; (**b**) implantation of the NeuroNexus probe (A1x16-3mm-100-177-CM16LP) in the rat cortical surface is shown here.

All surgical procedures, handling, and housing were approved by The University of Texas at Dallas Institutional Animal Care and Use Committee. Surgical procedures were performed as per previously established protocols [10,11,18]. Briefly, 9 Sprague-Dawley rats (6 males, 3 females; ~250 g) (Charles River Laboratories, Inc., Houston, TX, USA) were used. Animals were anesthetized with isoflurane (1.8–2.2%) (Covetrus North America, Dublin, OH, USA) supplemented with O_2_ and the head was shaved to expose the scalp. Toe pinches were used to confirm a deep anesthetized state and the rat was placed in a stereotactic frame (David Kopf Instruments, Tujunga, CA, USA). A far-infrared warming pad (Kent Scientific Corporation, Torrington, CT, USA) was placed below the animal to maintain the body temperature throughout the surgical procedure. Ophthalmic ointment (Lubrifresh P.M., Medline Industries, Wilmer, TX, USA) was applied to the eyes. After cleaning the scalp with alternating Betadine (Avrio Health L.P., Stamford, CT, USA) and alcohol swabs, marcaine (Hospira, Lake Forest, IL, USA) was injected under the scalp and an incision was made down the midline of the skull. Three stainless steel bone screws (Stoelting Co., Wood Dale, IL, USA) were inserted in the quadrants defined by bregma and the coronal and sagittal sutures to act as anchoring screws for the probe, as depicted in Figure 1a. As shown in Figure 1b, a 1 mm × 1 mm craniotomy was performed over the motor cortex (~2 mm from bregma and ~2 mm from central suture line) and dura mater was resected. A 16-channel, 3-mm-long probe (A1x16-3 mm-100-177-CM16LP, iridium electrode sites) (NeuroNexus Technologies, Ann Arbor, MI, USA) was implanted to a depth of 2 mm at a speed of 1 mm/s using a precision-controlled neural implantation device (NeuralGlider, Actuated Medical, Inc., Ann Arbor, MI, USA). Ground and reference wires from the neural probe were connected to two of the anchor screws. Collagen-based dural graft (Biodesign Dural Graft, Cook Medical, Bloomington, IN, USA) was placed in the craniotomy to cover the exposed cortical site. This was followed by a layer of GLUture (World Precision Instruments, Sarasota, FL, USA) to seal the craniotomy site. Once the GLUture cured, dental cement (Stoelting Co.) was used to form a head-cap that secured the probe to the anchoring screws and encapsulated the ground and reference wires of the probe. Surgical staples were utilized to close the midline incision and rats were administered 0.15 mL/kg of buprenorphine (ZooPharm, LLC., Laramie, WY, USA) and 0.05 mL/kg of cefazolin (Med-Vet International, Mettawa, IL, USA), and the antibiotic sulfamethoxazole and trimethoprin oral suspension (200 mg/40 mg/5 mL, Aurobindo Pharma, Dayton, NJ, USA) was added to the drinking water (1 mL/100mL drinking water) for a week post-surgery. After 72 h of surgery, animals received an additional dose of buprenorphine. 

### 2.2. Neurophysiogical Recording and Analysis

Data collection was performed from animal subjects approximately two weeks post-surgery and ten weeks post-surgery. Animals were anesthetized with isoflurane (inducted with 2.5%, maintained at 1.8–2.2%). Toe pinch was used to confirm anesthetized state of the animal, and a 10-minute recording was performed using an Omniplex recording system (Plexon Inc., Dallas, TX, USA). After completion of the anesthetized recordings, isoflurane was discontinued. The animals were allowed to recover after regaining consciousness and another 10-minute recording was performed while the rat was free to explore the recording cage. Continuous wide-band data were collected simultaneously from 16 electrode sites from both anesthetized and awake states for all nine animals in this study. Data were processed with low-cut and high-cut (300 Hz and 3000 Hz) 4-pole Butterworth filters, and digital common average referencing (Offline Sorter, Plexon Inc.) was used to eliminate local field potential contributions and common-mode noise. Individual spike waveforms were extracted using a -4σ threshold based on the root mean square (RMS) noise of the filtered continuous data [11,18]. Detected waveforms were then sorted for single units based on the 2D principal components analysis using a k-means sorting process with manual validation. 

From the obtained single-unit recordings, AEY was quantified as the proportion of total electrode sites exhibiting at least one well-distinguished single unit. For pairwise comparisons, only units that were observed and electrode sites that were active under both anesthetized and awake conditions were considered. The SNR was calculated as V_pp_ of a unit divided by the RMS noise of that channel. Since the MEA was implanted to a depth of 2 mm from the cortical surface, the device interacted with several cortical layers of the rodent brain. To investigate a depth-based analysis for the neural recording parameters, we classified the location of the 16 electrode sites as superficial (top 5 electrode sites), middle (central 5 electrode sites), and deep (the 6 electrode sites closest to the tip of the probe) regions, as per previously published work [18]. 

### 2.3. Statistical Analysis

OriginPro 2021 (OriginLab, Northampton, MA, USA) was utilized for statistical analysis. A test of proportions z-test was utilized to compare AEY across anaesthetized and awake states. The percent changes of SNR, V_pp_, RMS noise, and the change in mean firing rate were compared using one-sample Wilcoxon signed rank tests. Kruskal–Wallis ANOVA was utilized to compare depth-dependent changes (superficial, middle, and deep regions) in neural recording parameters. Data were considered significantly different at p < 0.05.

## 3. Results

To evaluate differences in recording metrics between isoflurane-anesthetized and awake conditions, we performed intracortical recordings from rats for 10 minutes under 1.8–2.2% isoflurane anesthesia, followed by another recording session once each animal subject regained consciousness and resumed movement.

For the earlier time point, single-unit activity was readily apparent on 73% of the electrode sites under anesthesia. The V_pp_ ranged from 32.7 to 499.5 µV, averaging 111.9 ± 62.8 µV (mean ± STD, n = 108). Across the implanted array, the single-unit firing rate on active channels spanned from 0.04 to 6.35 spikes/s, averaging 1.27 ± 1.67 spikes/s (mean ± STD, n = 108). In addition, the noise level during anesthesia ranged from 4.9 to 12.8 µV, averaging only 9.4 ± 1.7 µV_RMS_ (mean ± STD, n = 91), which resulted in SNR levels ranging from 6.3 to 56.0, averaging 11.9 ± 6.6 (mean ± STD, n = 108). 

**Figure 2:**
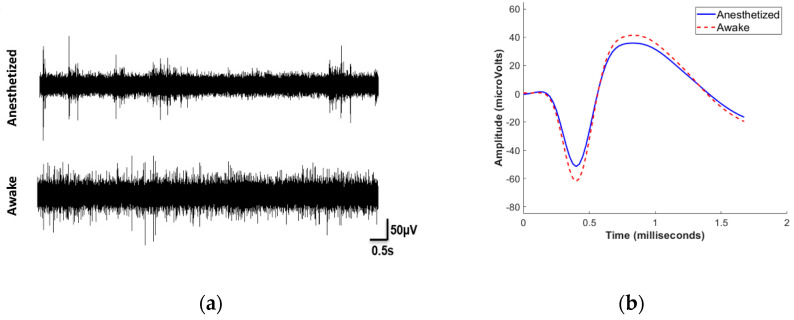
Neurophysiological data acquisition. (**a**) Exemplary single-unit recordings of an animal in anesthetized and awake state, respectively, are shown here. (**b**) Representative mean waveform of a unit under anesthesia and when awake.

**Figure 3:**
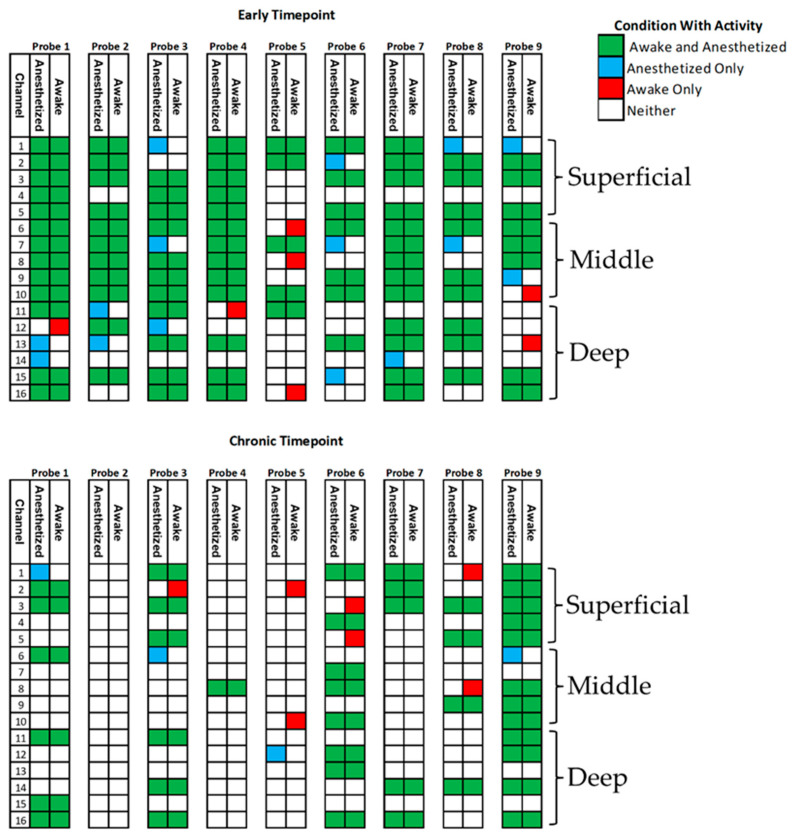
Effect of isoflurane anesthesia on AEY. Schematic depicting activity under anesthetized and awake conditions arranged by depth for early and chronic time points. The condition under which activity was observed is color-coded. Probe 2 was excluded from analysis at the chronic time point because units could not be reliably observed on the device by week 10.

With animal subjects recovering to the awake, freely behaving state, we observed a substantial increase in the spike firing rate, as illustrated by the representative single-channel recording in Figure 2a. The firing rate increased by 593 ± 104% (mean ± SEM, n = 108) when comparing awake to the anesthetized state. Given the role of the motor cortex in movement and consequent association with the awake state, the substantial increase in firing rate was not surprising. However, we did observe depth-dependent changes in the firing rate between the two conditions. When considering electrode sites by depth, we observed that units recorded on the superficial and deep sites increased significantly more than the middle sites on the arrays: superficial sites showed an elevation (p < 0.001) of 3.5 ± 0.5 spikes/s (mean ± SEM, n = 42), middle sites showed a less prominent increase (p < 0.05) of only 0.7 ± 0.3 spikes/s (mean ± SEM, n = 29), whereas the deep sites exhibited a marked rise (p < 0.001) of 5.5 ± 0.9 spikes/s (mean ± SEM, n = 37). As shown in Figure 2b, we observed only a slight, yet significant difference (p < 0.001) in V_pp_, which increased by 14.6 ± 2.2% (mean ± SEM, n = 108). Interestingly, the noise levels significantly increased (p < 0.001) by 46.9 ± 3.6% (mean ± SEM, n = 91) under awake conditions, which contributed to a statistically significant decrease (p < 0.001) in the SNR by 20.9 ± 2.1% (mean ± SEM, n = 108). Of critical importance for characterizing chronically implanted device reliability, we found little difference in the AEY under anesthetized and awake states. Although the AEY did not change, there was a small variation in the channels between the two recording conditions. Figure 3 summarizes the proportion of channels showing activity and the changes in the activity under the two conditions. Overall, 85.4% of recording channels exhibited no difference in activity (whether active or not) between the two conditions. However, 9.7% of the channels had detectable activity under anesthesia that was no longer detectable when awake. Only a small minority of recording channels, 4.9% of the total electrode sites, revealed activity under awake conditions that was not detected while the subject was under anesthesia. The activity under various conditions is summarized in Table 1.

Similar results were seen from the same animals at a chronic time point of 10–11 weeks, although the overall AEY was lower than the early time point. At this later time point, only 34% of electrode sites showed activity under anesthesia. Probe 2 was no longer able to record units and was left out of the analysis at the chronic time point. The frequency ranged from 0.08 to 4.10 spikes/s and averaged 1.17 ± 1.05 spikes/s (mean ± STD, n = 50). The V_pp_ ranged from 32.3 to 421.1µV, averaging 106.7 ± 74.0 (mean ± STD, n = 50). The noise levels were 3.6–13.4 µV_RMS_ and averaged 8.9 ± 2.2 µV_RMS_ (mean ± STD, n = 41), and the SNR ranged from 2.4 to 49.3 and averaged 12.5 ± 8.7 (mean ± STD, n = 50).

Recording metrics changed similarly at the chronic time point as they did at the early time point. The firing rate of units increased (p < 0.001) by 557% ± 143% (mean ± SEM, n = 50) when transitioning from anesthetic to the awake state. Unlike the recordings from the early time point, there was no discernable depth dependency to the change in frequency between the two conditions. The V_pp_ increase (p < 0.001) was slightly more pronounced at 20.0 ± 3.3% (mean ± SEM, n = 50), while the increase (p < 0.001) in noise was slightly less at 44.4 ± 5.5% (mean ± SEM, n = 41). These two changes caused a decrease (p < 0.001) in SNR of 11.3 ± 4.3% (mean ± SEM, n = 50), which is less prominent than the change in SNR at the early time point. Importantly, there was no significant change in the AEY between anesthetized and awake recordings at the chronic time point. Of the 128 electrode sites, 89.8% showed no change in activity—whether active or inactive—between the two conditions. There were 3.1% of the total channels that showed activity only under anesthesia, and 7.0% that showed activity only while awake.

## 4. Discussion

Our goal was to quantify the neurophysiological differences in single unit activity in the motor cortex of rats between conditions under anesthesia versus awake and freely behaving using intracortical silicon MEAs. We considered an early chronic period [7] post-implantation of MEAs and observed that the AEY was similar from these animals across the anesthetized and awake conditions. Furthermore, we noted a significantly higher SNR and lower noise levels for anesthetized recordings as compared to awake recordings. Our findings suggest that the use of isoflurane anesthesia is an acceptable methodology for studies assessing the reliability of intracortical MEAs, at least when implanted in the rodent motor cortex. Furthermore, these findings hold at both early and chronic time points.

Anesthetics such as isoflurane have a complex, reversible inhibitory effect on cortical activity and have a history of being considered safe and effective [22]. The inhibitory effect of isoflurane is dose-dependent [20,23] and involves suppression of burst activity [24,25]. Detsch et al. [23] found that increased dosages of isoflurane can change the response to stimuli-evoked activity in thalamocortical neuron populations. The changes occurred primarily after 1% and 2% isoflurane concentrations. Additionally, they saw an overall decrease in neuronal activity with an increase in isoflurane concentration, with no additional decrease in activity past 1.4% isoflurane. The change in activity is similar to the difference that we see in firing rates between awake and anesthetized recordings, and the level of isoflurane that we use is past the threshold of where they saw most of their differences. Others observed a bidirectional modulation of neural excitability [20] in mouse hippocampal brain slices. In that particular study, neural activity increased with subanesthetic levels of isoflurane. Under anesthetic concentrations of isoflurane, neural activity decreased. 

One concern of recording from anesthetized animals is that neurons may become quiescent under anesthesia, which could artificially lower the recorded AEY. A study by Noda et al. showed a loss of many units under isoflurane anesthesia (1.4–2.2%) in the auditory cortex of rats under isoflurane anesthesia (1.4–2.2%) and other modulation of single-unit activity and local field potentials [26]. When implanting over a large area at a single depth with a 96-channel Utah Array, they found that the total number of single units decreased under isoflurane anesthesia. However, they did not see a decrease in AEY under anesthesia, despite seeing fewer single units overall. The consistency of AEY under both conditions is consistent with what we have seen in our study. Although some unit activity was lost, the AEY remained the same. 

The lower noise levels due to the influence of isoflurane seemed to increase the SNR of the neural recordings in this study, which is useful to distinguish single units, especially those of smaller amplitude. The increased noise in awake recordings may be due to biological noise associated with a greater degree of neural activity under the awake state [27,28]. A study with mice implanted with silicon-based ATLAS Neuroengineering probes compared electrophysiological recordings from anesthetized (1% isoflurane), awake but resting, and running states of the animal, and found that running mode exhibited the highest level of noise as compared to the other two states [27]. Michelson et al. [29] utilized a planar, silicon-based, 16-channel microelectrode array, similar to our study, to compare the effect of isoflurane and ketamine on electrophysiology from awake or anesthetized mice. They observed no difference in the noise floor or the firing rate between anesthetized and awake conditions. However, they were using a probe similar to the one used in the present study but with larger electrode sites. Furthermore, recordings were performed from the visual cortex rather than the motor cortex. More importantly, they were using a minimal dose of isoflurane (approximately 1%) to maintain an anesthetized state while avoiding burst suppression. The dose-dependent effects of isoflurane noted by Ou et al. [20] may contribute to some of the differences between our studies. Moreover, in agreement with the study from Ou and colleagues, we observed a lower V_pp_ under anesthesia than awake recordings. This is possibly due to the increase in leak conductance caused by isoflurane [24]. Furthermore, Ou et al. showed an increase in sodium channel leak currents caused by isoflurane. This could lead to a higher intracellular concentration of sodium, resulting in decreased action potential amplitudes [30], as seen in both our study and the Ou et al. study. Another potential contribution to the noise increase under awake conditions may be due to motion artifacts that occur during awake recordings. Motion artifacts can potentially mask the signals from single units, but with filtering and common average referencing, they may also appear more “unit-like” in amplitude and shape [31].

Since the probe was implanted to a depth of approximately 2 mm, the MEA interacted with different layers of the rodent cortex. At the early time point, we observed depth-dependent changes in firing rates between electrodes located in the superficial, middle, and deep regions, respectively. We observed greater increases in the firing rate after removal of anesthesia in the upper and lower electrode sites than the middle electrode sites. The overall observations for awake versus anesthetic state hold at a chronic time point. However, due to the loss of active recording sites under chronic implantation conditions, it becomes difficult to statistically distinguish differences in firing rate due to depth dependence. Isoflurane has been shown to affect the response to stimuli across layers differently and to increase intralaminar LFP coherence in the visual cortex of mice [29]. Another possible explanation for the increased firing rate, at least for the deep microelectrode sites, includes a tendency of units in the corresponding layers of the motor cortex to increase the firing rate with respect to movement [32]. It is possible that the differences in the density and diversity of neuronal cells present in different layers of the rat cortex [33] could lead to the variation in firing rate across the cortical layers. Taken together, the data suggest that isoflurane-based anesthetized recordings provide us with well-resolved single unit activity, without compromising determination of electrode functionality, as compared to recordings from the same animals in the awake state. 

## 5. Conclusions

We have systematically compared single-unit recording waveforms, frequency, and yield under isoflurane-anesthetized and awake conditions for implanted microelectrode arrays within the rat motor cortex. Our findings indicate that while the frequency of single-unit firing is much higher for the awake state, there is a small improvement in SNR and virtually no difference in the AEY under isoflurane anesthesia. Regarding the changes in frequency, firing rates between anesthetized and awake recordings appeared dependent on the implantation depth within the motor cortex, at least for the early recording time point. Our findings suggest that there are few drawbacks to using isoflurane anesthesia when assessing the chronic performance and reliability of intracortical microelectrode arrays using common metrics of noise levels, SNR, and AEY. If the primary goal of a study is to assess probe function, anesthetizing the animal before and during recordings can make handling the subjects easier. In addition, anesthetized recordings show either a slight improvement or no difference in the metrics of interest when compared to recordings in the same animals while awake. These observations suggest that it is acceptable to assess in vivo chronic MEA reliability and performance for devices implanted in the rodent motor cortex. 

## Figures and Tables

**Table 1:** AEY of anesthetized and awake conditions for early and chronic time points.

Condition Showing Activity	AEY (%) at Early Time Point (n = 9)	AEY (%) at Chronic Time Point (n = 8)
Anesthetized and Awake	63.2	31.3
Anesthetized Only	9.7	3.1
Awake Only	4.9	7.0
Neither	22.2	58.6
